# Comprehensive analysis of the basement membrane in lung adenocarcinoma by bulk and single-cell sequencing analysis

**DOI:** 10.7150/jca.83407

**Published:** 2023-06-04

**Authors:** Hanyu Shi, Liang Sun, Bin Liu

**Affiliations:** 1 Department of Internal Medicine, Hospital of the First Mobile Corps of the Chinese People's Armed Police Force, Dingzhou, Hebei, 073099, China.; 2 Department of Pulmonary and Critical Care, Characteristic Medical Center of the Chinese People's Armed Police Force, Tianjin, 300162, China.

## Abstract

**Background:** The basement membrane (BM), as a critical component of the extracellular matrix, plays a role in cancer progression. However, the role of the BM in lung adenocarcinoma (LUAD) remains unclear.

**Methods:** A total of 1383 patients from The Cancer Genome Atlas (TCGA) and Gene Expression Omnibus (GEO) cohorts were enrolled in the study, and BM-related differentially expressed genes (BM-DEGs) were screened using weighted gene coexpression network analysis (WGCNA) and differential expression analysis. We next built a prognostic model using Cox regression analysis and separated patients into two groups based on the median risk score. This signature was validated with in vitro experiments, and its mechanism was investigated by enrichment and tumour microenvironment analyses. We also evaluated whether this signature could predict sensitivity to chemotherapy and immunotherapy. Finally, single-cell RNA sequencing analysis was utilized to analyse the expression of signature genes in different cells.

**Results:** Thirsty-seven BM-DEGs were discovered, and a prognostic signature based on 4 BM-DEGs (*HMCN2*,* FBLN5*, *ADAMTS15* and *LAD1*) was obtained in the TCGA cohort and validated in GEO cohorts. Survival curves and ROC curve analysis demonstrated that the risk score was a significant predictor of survival in all cohorts even when considering the effect of other clinical indexes. Low-risk patients had longer survival times, higher immune cell infiltration levels and better immunotherapeutic responses. Single-cell analysis showed that *FBLN5* and *LAD1* were overexpressed in fibroblasts and cancer cells, respectively, compared to normal cells.

**Conclusion:** This study evaluated the clinical role of the BM in LUAD and primarily explored its mechanism.

## Introduction

Lung cancer, the second most common malignant neoplasm in 2022, has the highest mortality rate among all malignancies worldwide 1. Non-small cell lung cancer (NSCLC) accounts for approximately 85% of all lung cancer cases, and lung adenocarcinoma (LUAD) is the most common histological subtype, accounting for 47% of NSCLC cases 2. Despite substantial advances in treatment approaches, the overall prognosis of LUAD patients remains poor, so increasing emphasis has been placed on the discovery of novel potential molecular targets to facilitate early diagnosis and effective treatment, thus improving the overall prognosis 1, 3, 4.

The basement membrane (BM) is a special type of extracellular matrix (ECM) produced by epithelial and endothelial cells. Recent findings support that BM plays a critical role in resistance to mechanical stress, construction of a diffusion barrier, and promotion of cell polarity and differentiation 5, 6. The major constituents of the BM include laminin, collagen, nidogen and heparan sulfate proteoglycan 7. Approximately 66-90% of cancer patients die of cancer metastasis, which is triggered by the penetration of tumour cells through the BM 5. Therefore, it is essential to evaluate the clinical role of the BM in LUAD and investigate its potential mechanism. Despite the fact that models based on BM-mRNAs (messenger RNA) have been developed, our research was conducted to identify more practical models and investigate the therapeutic response (particularly for targeted drugs) from additional aspects 8, 9.

In this study, BM-related data of LUADs from public databases were analyzed to construct a prognostic model and validated with in vitro experiments. Moreover, we evaluated the ability of the model to predict immunotherapy efficiency and explored the potential mechanism.

## Methods

### Data collection and processing

The mRNA data of LUAD samples and corresponding clinical information were downloaded from The Cancer Genome Atlas portal (TCGA, https://portal.gdc.cancer.gov, n=535, training cohort) and Gene Expression Omnibus database (GEO, https://www.ncbi.nlm.nih.gov/gds, dataset IDs: GSE72094, GSE68465, n=862, external validation cohorts) on August 10, 2022. Patients were eligible to enroll if they met the following requirements: (1) their histological diagnosis was LUAD; and (2) data on the gene expression and clinical features were available from samples. The exclusion criteria were as follows: (1) initial histologic diagnosis was not LUAD; and (2) presence of malignant neoplasms apart from LUAD. Finally, 1383 eligible patients participated in this study. In addition, single-cell sequencing data of 11 primary LUADs (GSE131907) were selected to explore the role of the BM signature between cells. The baseline data for all tumour patients are reported in **Table S1**.

### Screening for BM-related differentially expressed genes (BM-DEGs) by differential analysis and WGCNA

Previous research revealed that a total of 224 BM-related genes (**Table S2**) are involved in tumour development 10. With normal tissue as a control, we performed differential expression analysis of TCGA-LUAD samples with the Wilcoxon test using the “limma” R package. Genes with an adjusted *P* value<0.05 and | log2(fold change) | > 1 were selected. Weighted gene coexpression network analysis (WGCNA) was also performed to mine the core genes using the “WCGNA” package 11. Significant modules most relevant to LUADs were selected as target modules, and genes within them were chosen. The differentially expressed genes that overlapped with the two algorithms were defined as BM-DEGs.

### Construction and evaluation of the prognostic model based on BM-DEGs

All BM-DEGs were included in the univariate Cox regression model, and those with P values <0.05 were included in the multivariate Cox model to select prognostic BM-DEGs (BMGs) using the “survival” package. The risk score formula based on the multivariate Cox model was as follows: (expression of A * coefficient of A + expression of B * coefficient of B+……+ expression of N * coefficient of N). Survival curves were plotted using the Kaplan-Meier method with the “survminer” package. Time-dependent receiver operating characteristic curves (t-ROCs) were drawn to predict the accuracy of the model using the “timeROC” package. In addition, we compared the expression levels of BMGs between groups and assessed their correlations with the risk score. Finally, a nomogram was generated with the “rms” package and assessed by calibration curves and decision curve analysis (DCA). External validation was performed on two GEO validation cohorts.

### External validation by quantitative real-time polymerase chain reaction (qRT‒PCR) and western blotting (WB)

BMG expression was compared between human normal bronchial epithelial cells (Beas-2B) and human LUAD cell lines (A549). Cell lines with a good growth status were selected for further analysis, and each test was conducted in triplicate to determine the average. The results of qRT‒PCR and WB were analysed using 7500 System Software V2.3 (Applied Biosystems, CA, USA) and ImageJ software (version 2.1.4.7 (National Institutes of Health), respectively. The detailed protocols for the in vitro experiments are provided in the **Supplemental Materials**.

### The therapeutic response to chemotherapy and immunotherapy

Individual chemotherapy sensitivity was estimated by the “oncoPredict” package, and the Wilcoxon test was applied to compare the difference in IC50 (half maximal inhibitory concentration) between groups 12. Apart from the expression of immune checkpoint inhibitors (ICIs: PD-1, PD-L1, CTLA4, HAVCR2, LAG3, TIGIT) 13, 14, the tumour mutation burden (TMB) and T-cell receptor (TCR) repertoire were also found to predict immunotherapy efficiency 15, 16. Additionally, patients from the IMvigor210 and GSE78220 cohorts with both sequencing data and immunotherapy response data were selected to evaluate the ability of the signature to predict immunotherapy efficiency 17.

### GSEA and GSVA based on BMGs

To explore the potential mechanism of the BMG-related signature, GO and KEGG gene set enrichment analyses (GSEA) were performed using GSEA tools (v4.2.1, http://www.broadinstitute.org/gsea), and the results were visualized with the “ggplot2” package. The top 5 enriched GO and KEGG items with adjusted P values<0.05 in the high-risk group and low-risk group were selected and are shown. We also performed gene set variation analysis (GSVA) using the “GSVA” package 18 to compare enriched pathways between groups. The above gene sets were downloaded from the MSigDB database (https://www.gsea-msigdb.org/gsea/msigdb).

### Tumour microenvironment (TME) characterisation using immune cell infiltration analysis and the ESTIMATE algorithm

The single-sample GSEA (ssGSEA) algorithm was adopted to calculate the scores of infiltrating immune cells and functions between groups in the TCGA and GSE72094 cohorts 18. Next, we calculated the stromal score (representing the infiltration levels of stromal cells), immune score (representing the infiltration levels of immune cells), ESTIMATE score (reflecting the cell infiltration degree in the TME) and tumour purity in each sample using the “ESTIMATE” package 19, 20.

### Evaluation of BMGs in LUADs by single-cell RNA (scRNA) sequencing analysis

To explore the role of the signature in different cells, we conducted scRNA analysis using the “Seurat” package 21. We calculated the expression of BMGs in each cell type and compared the cell proportion between risk-based groups. GSVA was carried out to evaluate the enrichment score of the pathways at single-cell resolution. Furthermore, we analyzed cell‒cell interaction using the “CellChat” package (https://github.com/sqjin/CellChat/) based on the expression of known ligand‒receptor pairs in different cell types 22. The detailed protocol is presented in the **Supplemental Materials.**

### Statistical analysis

**Figure 1:**
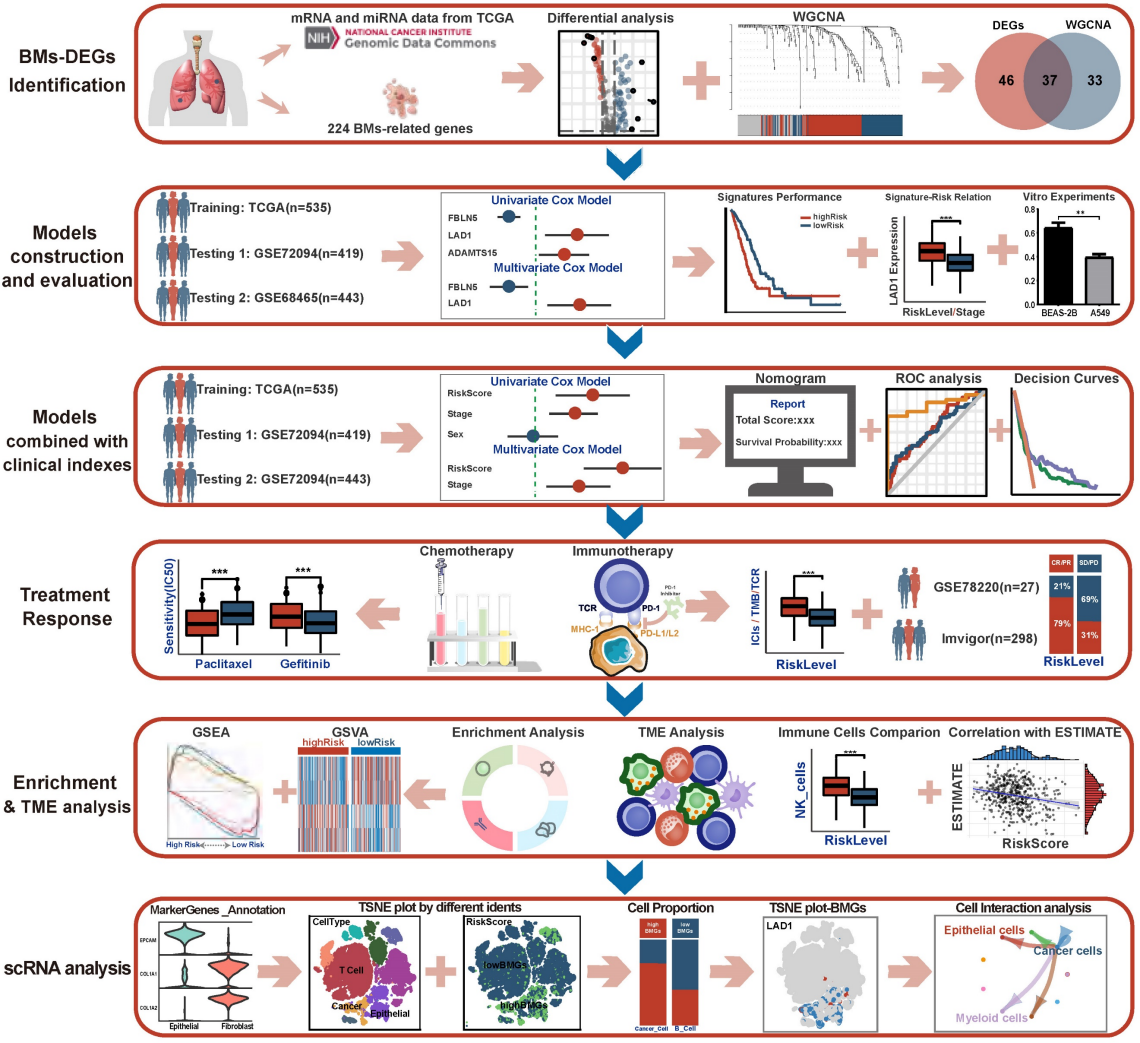
The framework and flowchart of this study.

All data processing, statistical analysis and graph plotting were performed with R software (v4.0.1, https://www.r-project.org/). A two-tailed *P*<0.05 was considered to indicate statistical significance (* *P*<0.05, ** P<0.01, *** *P*<0.001, ns* P*>0.05). The framework and workflow of this study are summarized in **Figure 1.**

## Results

### Thirty-seven BM-DEGs were identified by WGCNA and differential expression analysis

**Figure 2:**
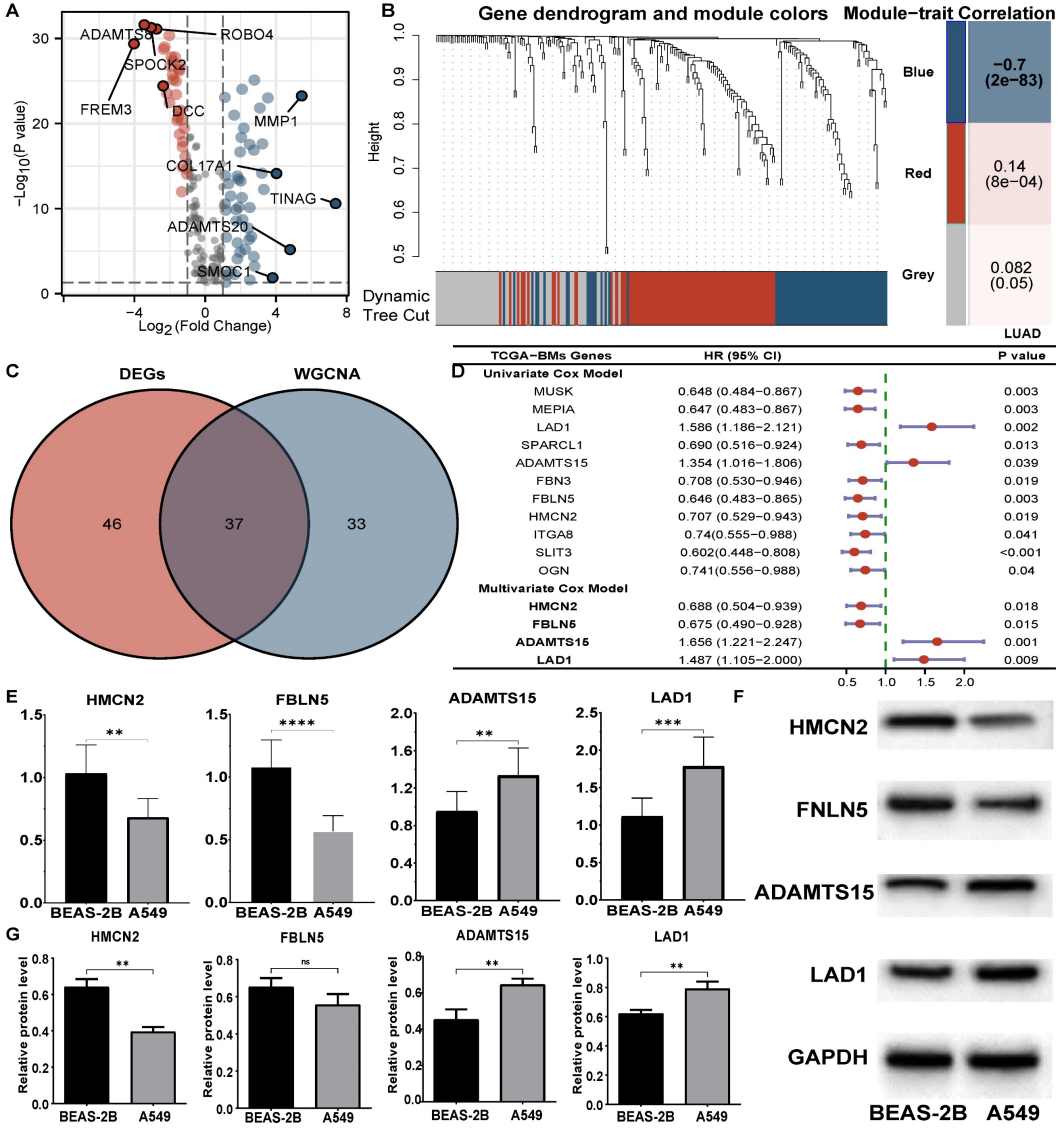
Identification of prognostic BM-DEGs in LUADs. (A) Volcano plots of differential analysis. (B) Gene dendrogram obtained from WGCNA and module-patient trait relationships plot. (C) Venn diagram of BM-DEGs from two methods. (D) Forest plot of univariate and multivariate Cox analysis of BM-DEGs. (E) RT‒PCR results of BMGs. (F-G) The WB bands and analysis of BMGs. ** *P*<0.01, *** *P*<0.001, ns *P*>0.05.

The expression profiles of tumour and normal tissues were compared, and 83 of 224 BM-related genes were screened (**Figure 2A, Table S2**). According to the scale independence and average connectivity of WGCNA networks (**Figure S1**), a power value of 4 was chosen as the best soft threshold power. The results suggested that the blue module (R^2^=-0.70,* P*=2e-83) was most significantly correlated with LUAD status (LUAD vs. normal), and 70 genes were chosen from this module (**Figure 2B, Table S2**). In total, 37 genes overlapping between the two methods (**Figure 2C, Table S2**) were identified as BM-DEGs for further analysis.

### A four-gene prognostic signature was built and evaluated in the TCGA cohort

Eleven BM-DEGs with prognostic roles (**Figure 2D**) were discovered by univariate Cox models and entered into multivariate analysis. As a result, four genes (**Figure 2D**) revealed a significant role in predicting the prognosis of LUADs and were identified as BM-related prognostic genes (BMGs). The risk score formula was as follows: risk score=0.688*HMCN2+0.675 *FNLN5+ 1.656*ADAMTS15+1.487 *LAD1. The qRT‒PCR results suggested that *HMCN2* and *FBLN5* were downregulated in A549 cells, while *ADAMTS15* and *LAD1* were upregulated (**Figure 2E**). Moreover, the results of WB were consistent with these findings (**Figure 2F, G**).

**Figure 3:**
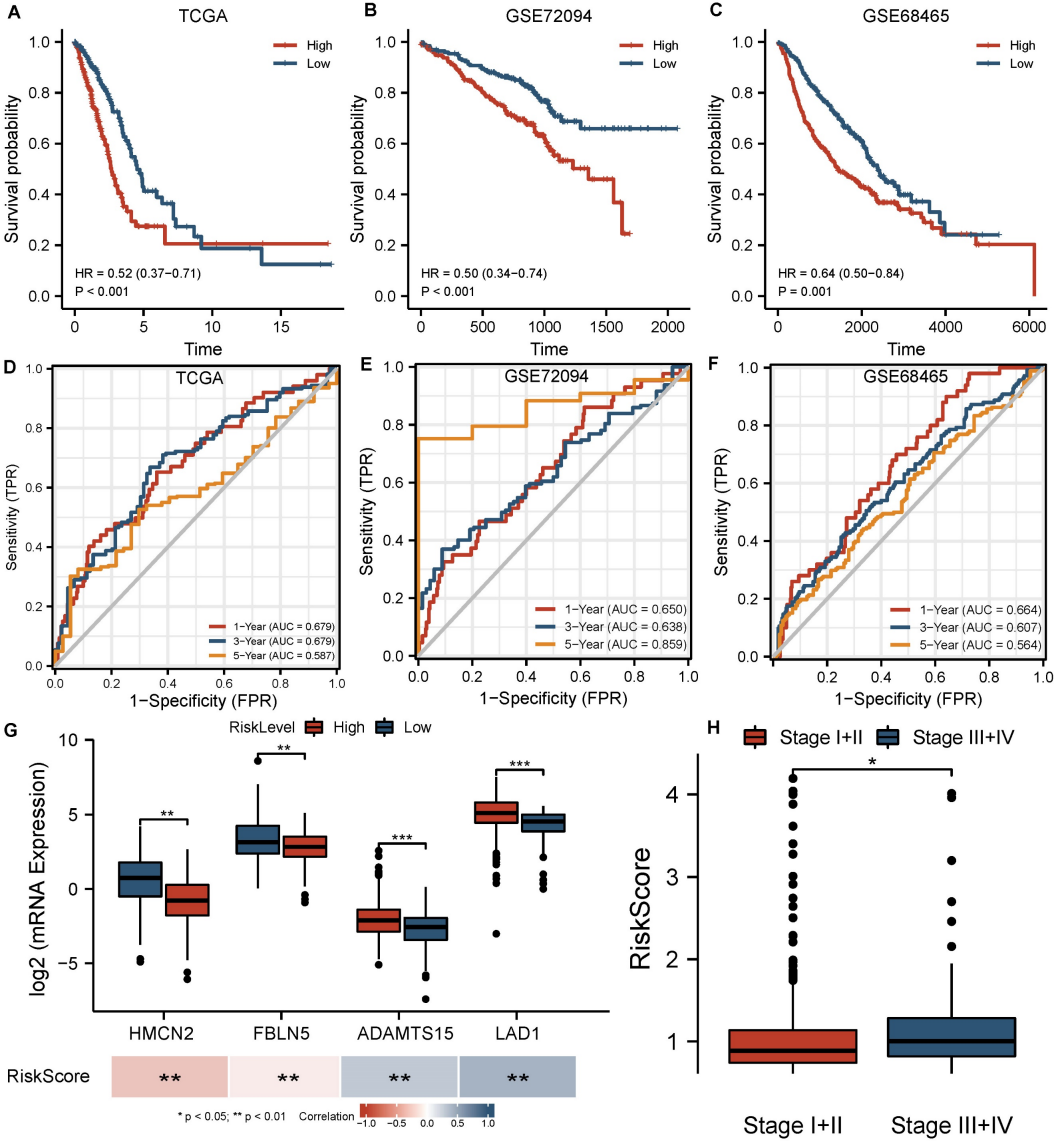
Evaluation of BMGs in the training and validation cohorts. (A-C) Survival curves based on the BMG-related risk in TCGA, GSE72094, GSE68465 cohorts. (D-F) Time-independent ROC curves based on the BMG-related risk in TCGA, GSE72094, GSE68465 cohorts. (G) Comparisons of BMG expression between groups and their association with risk score. (H) Comparisons of BMG-related risk score between stages. * *P*<0.05, ** *P*<0.01, *** *P*<0.001, ns *P*>0.05.

All patients in the TCGA cohort were classified into high-risk and low-risk groups based on the median risk score from the 4-BMG signature. The survival curve indicated that high-risk patients had a lower overall survival rate (HR=0.52, *P*<0.001, **Figure 3A**). The areas under the ROC curves for 1, 3, and 5 years were 0.679, 0.679, and 0.587, respectively (**Figure 3D**). In addition, substantial differences in the expression of the four BMGs were identified between risk groups, and the levels of all these BMGs were significantly associated with the risk score (all *P*<0.001,** Figure 3G**). A high tumour stage implied a higher risk score (*P*<0.05, **Figure 3H**). Otherwise, there were no significant differences between the age and sex groups (**Figure S2**).

### The signature remained a strong predictive factor when clinical variables were incorporated

**Figure 4:**
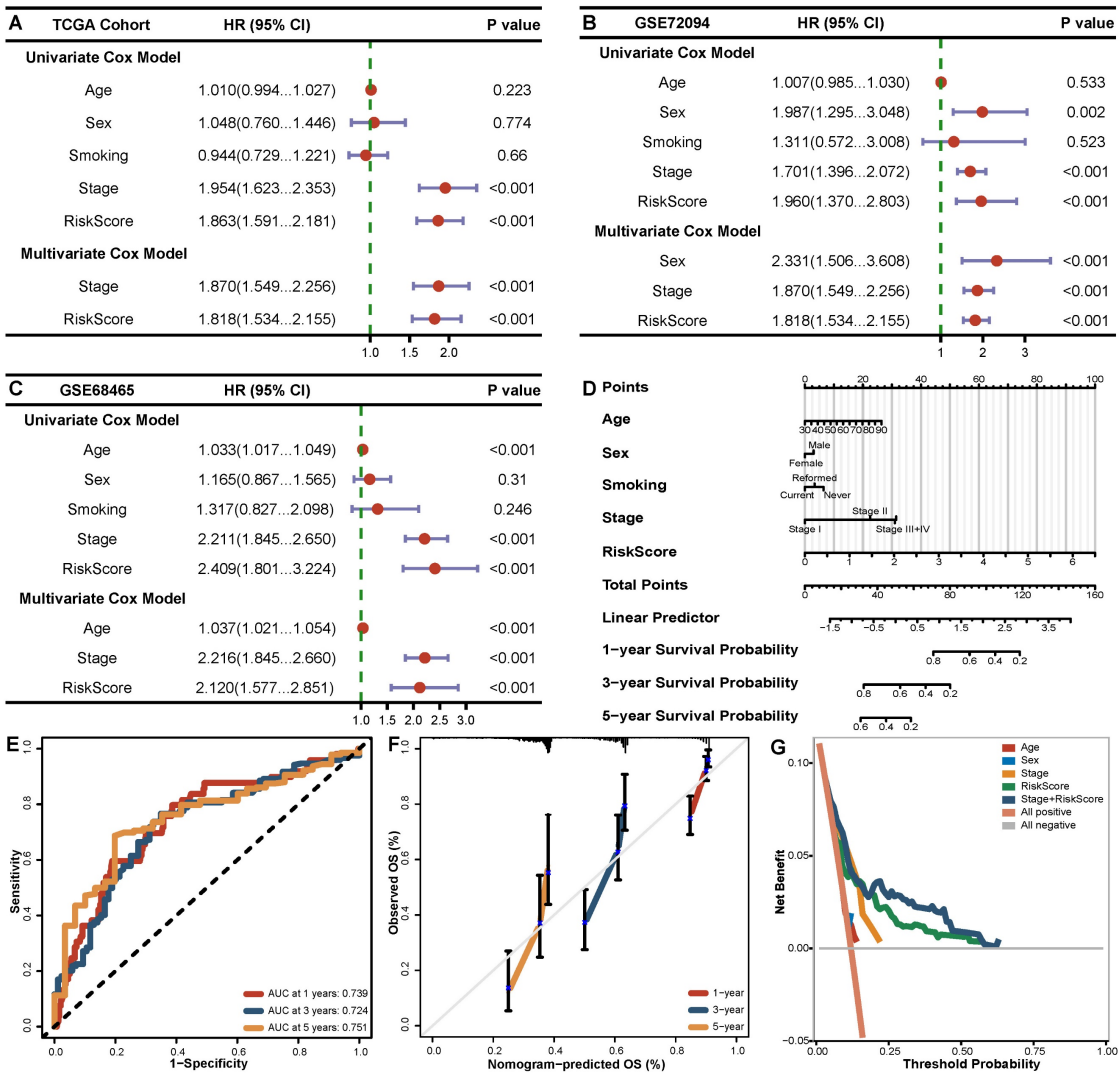
The independent prognostic role of BMGs in LUADs. (A-C) Forest plot of univariate and multivariate Cox analysis of BMGs combined with clinical indexes in TCGA, GSE72094, GSE68465 cohorts. (D) Nomogram for the overall survival of LUAD patients in the TCGA cohort. (E-G) ROC curves, calibration curves and decision curves of the nomogram model.

The next section of our study was concerned with the prognostic value of the signature when the model incorporated clinical features (age, sex, smoking, stage). Multivariate Cox analysis showed that only stage (HR=1.870, 95% CI 1.549-2.256, *P*<0.001) and risk score (HR=1.818, 95% CI 1.534-2.155, *P*<0.001) were independent predictors in the TCGA training cohort (**Figure 4A**). Due to the noticeable predictive value of the risk score, we integrated it with clinical features to create a nomogram to realize a more practical clinical application strategy (**Figure 4D**). The AUC values of the nomogram reached 0.739, 0.724 and 0.751 for 1, 3 and 5 years, respectively (**Figure 4E**). The calibration curves at 1, 3 and 5 years presented excellent consistency with the actual observations (**Figure 4F**). Moreover, the results from DCA demonstrated better discriminatory power of the comprehensive model compared to traditional clinical indexes (**Figure 4G**).

### External validation of the signature in GEO cohorts

A significantly lower survival rate was observed in the high-risk groups in both GEO validation cohorts (GSE72094 HR=0.50, *P*<0.001; GSE68465 HR=0.64, *P*=0.001) (**Figure 3B, C**). The AUCs for the validation cohorts ranged from 0.650-0.664, 0.607-0.638 and 0.564-0.859 for 1, 3 and 5 years, respectively (**Figure 3E, F**), denoting decent predictive value in all cohorts. The Cox regression analysis including the risk score and clinical features (**Figure 4B, C**) suggested that the risk score (HR 1.818-2.120 *P*<0.001) and stage (HR 1.870-2.216, *P*<0.001) were significantly predictive of patient outcome in the two GEO cohorts.

### High-risk LUAD patients presented higher sensitivity to target drugs, while low-risk LUAD patients were more sensitive to immunotherapy

**Figure 5:**
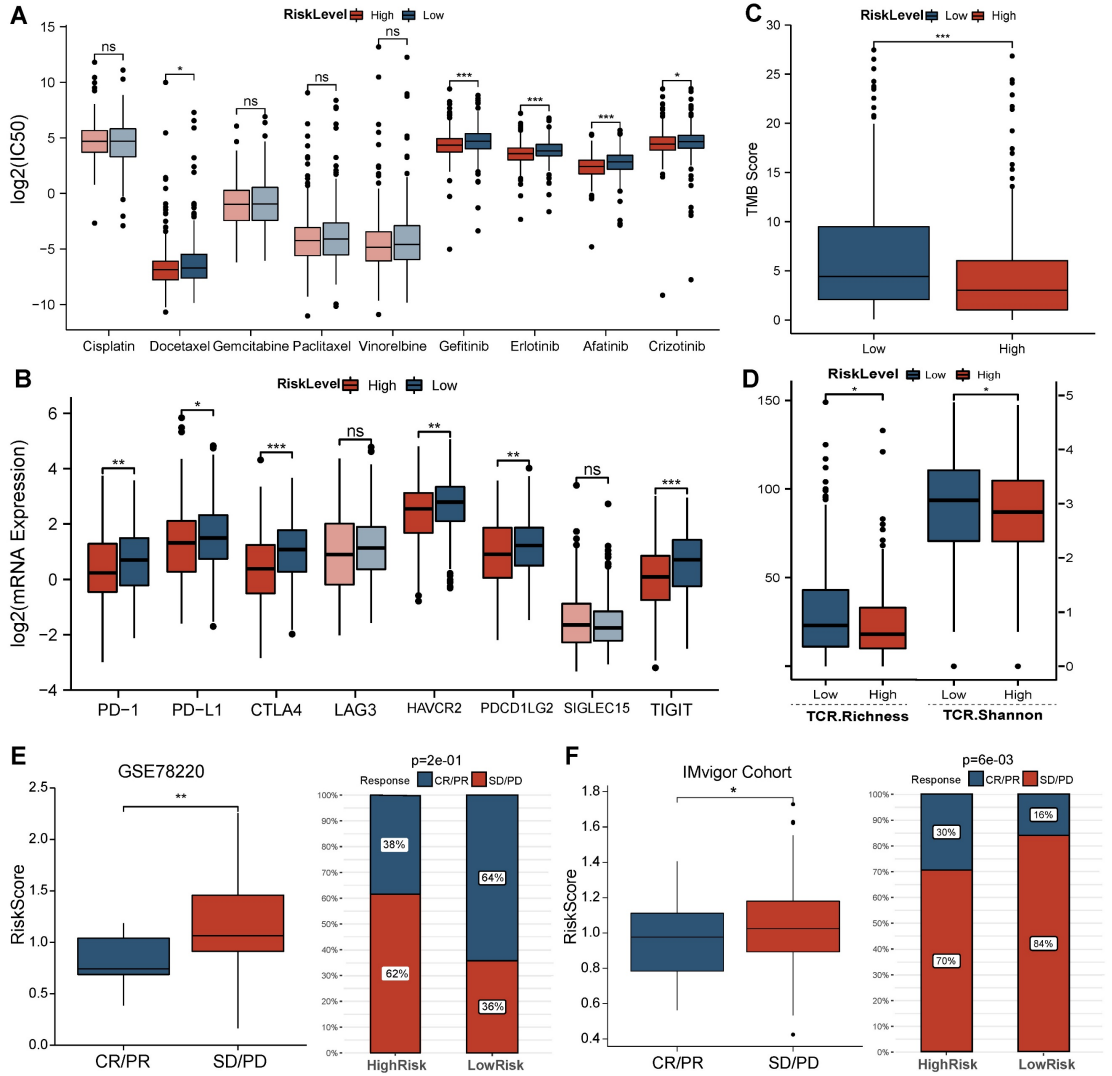
Predictions regarding the efficacy of chemotherapy and immunotherapy. (A) Comparison of sensitivity of common agents administered in LUADs between groups. (B-D) Comparison of ICI target expression, TMB and TCR repertoire between groups in TCGA cohort. (E-F) Comparisons of BMG-related risk score between responder and nonresponder groups in GSE78220 and IMvigor210 cohorts, the proportion of patients with each response type between BMG-related groups. * *P*<0.05, ** *P*<0.01, *** *P*<0.001, ns *P*>0.05.

Comparison of the sensitivity of LUAD patients in different risk groups to common agents (**Figure 5A**) revealed that high-risk LUAD patients presented high sensitivity to the traditional chemotherapeutic agent docetaxel (*P*<0.05) and multiple target drugs such as gefitinib, erlotinib, afatinib, and crizotinib (all *P*<0.05). Regarding the immunotherapy response, the expression of multiple ICI targets in the low-risk group was clearly enhanced (PD-1 *P*<0.001, CD274/PD-L1 *P*<0.001, CTLA4 *P*<0.001, PDCD1 *P*<0.01, TIGIT *P*<0.01, HAVCR2 *P*<0.001, **Figure 5B**). These results primarily suggest that LUAD patients with a low risk score are more sensitive to ICIs than those with a high risk score. In addition, we observed a higher TMB in low-risk LUAD patients and the best overall survival rate in the high TMB plus low-risk group (**Figure 5C, Figure S3**). The higher TCR richness and diversity also confirmed these findings (**Figure 5D**). Furthermore, the results from immunotherapy-treated cohorts (**Figure 5E, F**) demonstrated that responders (complete response/partial response, CR/PR) exhibited lower risk scores (GSE78220 *P*<0.01, Imvigor210 *P*<0.05) than nonresponders (stable disease/progressive disease, SD/PD). The response rate to anti-PD-L1 therapy was also markedly elevated in the low-risk group (GSE78220 *P*<0.001, Imvigor210 *P*<0.001).

### GSEA and GSVA enrichment analysis based on BM-DEGs

**Figure 6:**
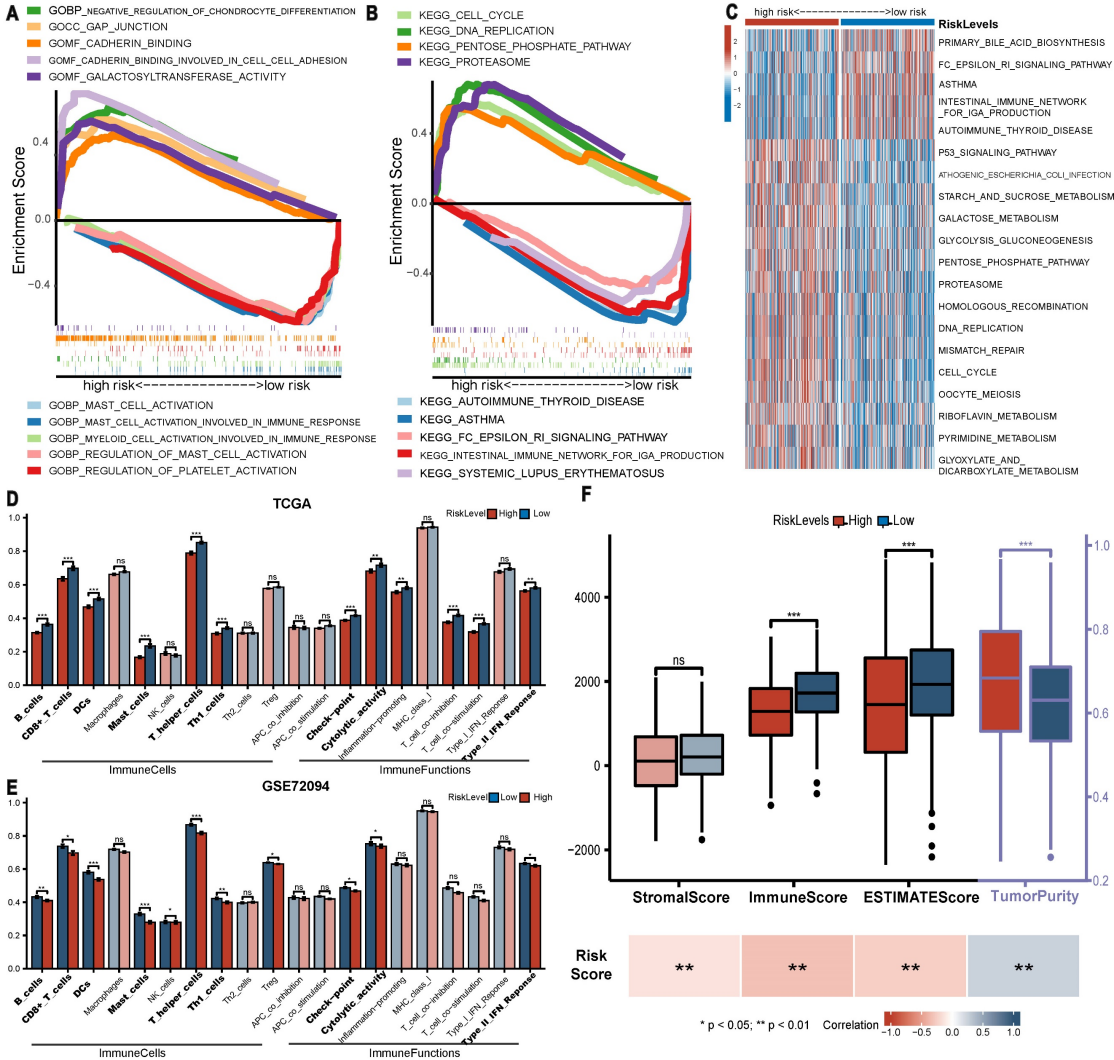
Enrichment analysis and TME analysis. (A-B) GO and KEGG gene set enrichment analysis by GSEA. (C) Heatmap displaying the results of GSVA analysis. (D-E) Comparison of the ssGSEA scores of immune cells and functions between groups in the TCGA and GSE72094 cohorts. (F) Comparison of ESTIMATE score between groups and their association with risk score. * *P*<0.05, ** *P*<0.01, *** *P*<0.001, ns P>0.05.

Regarding enrichment results by GSEA, GO analysis (**Figure 6A**) revealed that gap junction, cadherin binding and galactosyltransferase activity were enriched in the high-risk group, while mast cell activation, myeloid cell activation involved in immune response and regulation of platelet activation were involved in the low-risk group. The KEGG analysis suggested that pro-oncogenic pathways (cell cycle, DNA replication, proteasome and pentose phosphate pathway) were enriched in the high-risk group. Immune-related pathways (autoimmune thyroid disease, asthma and systemic lupus erythematosus) were enriched in the low-risk group (**Figure 6B**). Moreover, the GSVA results confirmed the above findings (**Figure 6C**). These findings largely clarified the potential mechanism underlying the observation of worse survival in the high-risk group.

### Low-risk LUAD patients presented greater immune infiltration

Significant differences were observed in multiple immune cells and immune-related functions between groups (**Figure 6D, E**). Specifically, LUADs with low risk scores exhibited definitively higher levels of 6 immune cells (B cells, CD8+ T cells and. etc., *P*<0.01) in both the TCGA and GSE72094 cohorts. Moreover, in both cohorts, low-risk LUADs had higher checkpoint molecule expression, cytolytic activity and type II IFN response levels, which are all critical targets in the antitumour biological process. Further analysis via ESTIMATE (**Figure 6F**) revealed that the immune and ESTIMATE scores of LUADs were clearly higher in the low-risk group and correlated negatively with the risk score, and tumour purity was higher in the high-risk group. However, no significant difference in stromal score was observed in the high and low risk groups.

### The expression of signature genes is increased in cancer cells and associated with stronger cell-cell interactions

**Figure 7:**
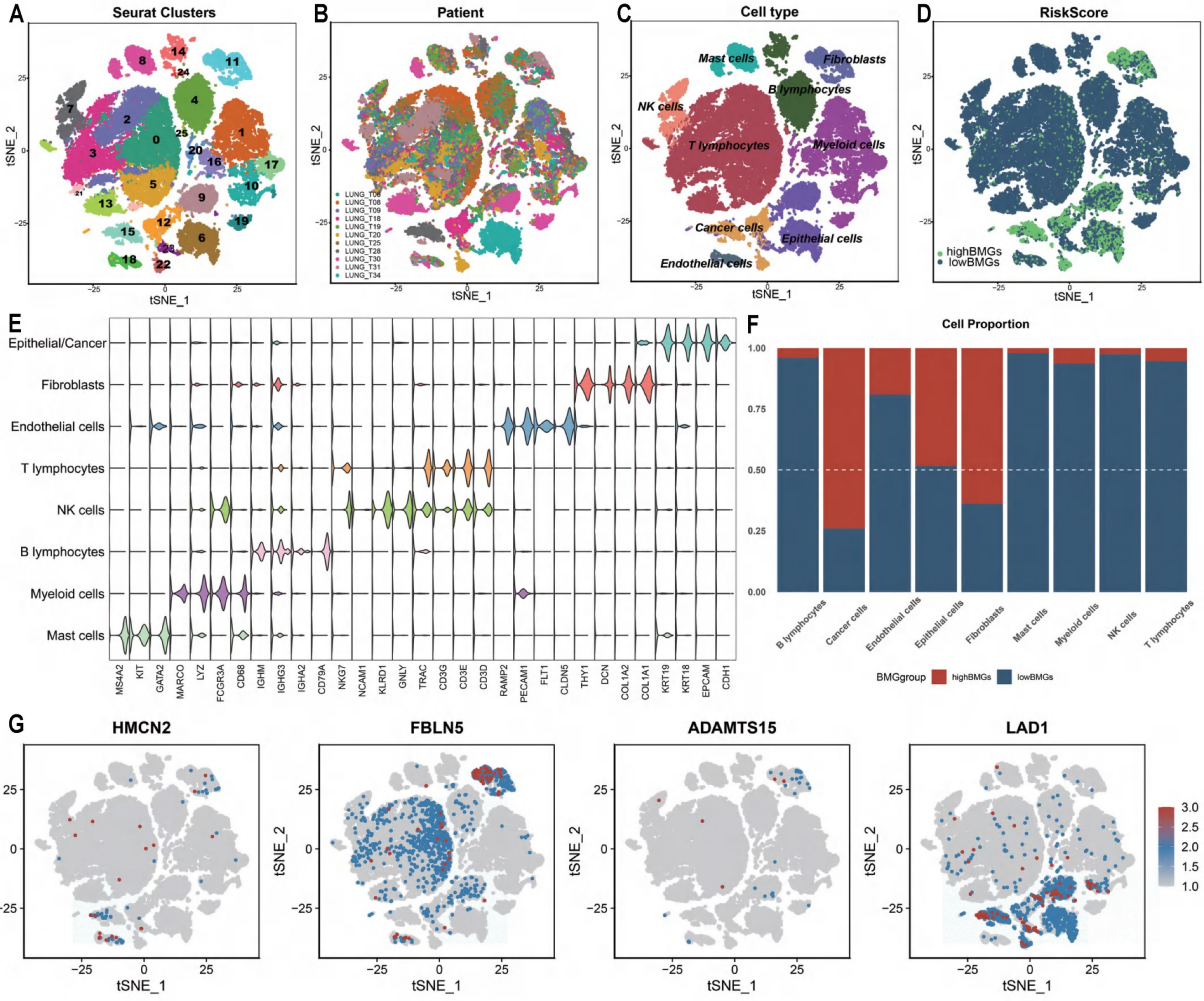
Evaluation of BMGs between cells by scRNA-seq analysis. (A-D) TSNE plot grouped by clusters, patients, cell type and risk group. (E) Violin plot displaying marker genes of each cell type. (F) The proportion distribution of different cells between BMG-related groups. (G) The distribution of BMGs across the different cells by TSNE plot.

**Figure 8:**
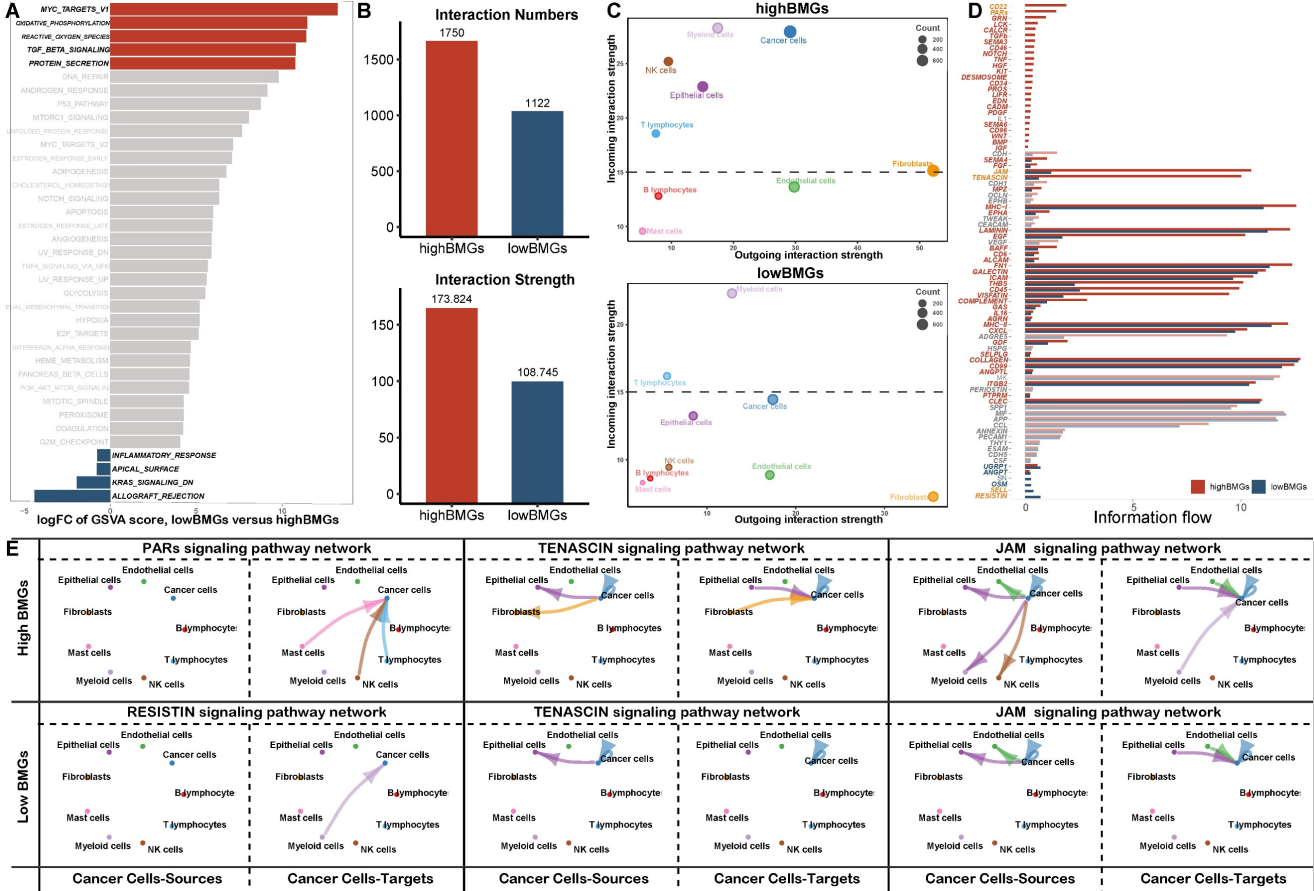
GSVA and cell‒cell interactions according to scRNA-seq analysis. (A) Boxplot displaying GSVA results at single-cell resolution. (B-D) Comparison of the total number and strength of interactions, the outgoing and incoming interaction strength in 2D space and the overall information flow for the top signalling pathways between BMG-related risk groups. (E) Circle plots shown the interactions between cancer and other cells in top signalling pathways.

With the previously mentioned protocol, a total of 44196 LUAD cells (high BMG expression: 7056 cells, low BMG expression: 37140 cells) passed quality control, and 26 distinct clusters were identified (**Figure 7A**). The t-distributed stochastic neighbour embedding (TSNE) plot was used to cluster patients and suggested that there were no batch effects between samples (**Figure 7B**). We performed integrated annotation (**Figure 7C**) on the basis of marker genes (**Figure 7E**) and identified 2347 cancer cells from epithelial cells using the “copykat” algorithm (**Figure S4**). The samples consisted of cancer cells, epithelial cells, fibroblasts, etc. As shown in **Figure 7D and Figure 7F**, samples with high BMG expression had a higher proportion of cancer cells and fibroblasts than those with low BMG expression. *FBLN5* was notably upregulated in fibroblasts, and *LAD1* was markedly upregulated in cancer cells (**Figure 7G**). GSVA at the single-cell level also showed that low BMG expression was associated with the inflammatory response and downregulation of the pro-oncogenic KRAS signalling pathway (**Figure 8A**).

Next, we evaluated the interactions between cells with different BMG expression levels. The total number of interactions and the strength of interactions were significantly elevated in cells with high BMG expression (**Figure 8B, Figure S5**), and the interaction difference was most prominent among cancer cells, epithelial cells and fibroblasts (**Figure 8C, Figure S6**). Furthermore, we explored the most robust interactions, especially those involving cancer cells. Our results showed that cancer cells at high BMG levels mainly communicate with other cells via the PAR, TENASCIN and JAM pathways, thereby promoting tumour metastasis and progression (**Figure 8D, E, Figure S7**).

## Discussion

Immunotherapy with ICIs is the most recent advancement and has been most revolutionary treatment for LUAD 23. Nevertheless, in unselected patient populations, only a limited proportion of LUAD patients benefit from ICIs; thus, it is imperative to screen patients who may benefit from immunotherapy 1, 3, 4, 24. Previous research has shown that BMs are significantly related to the advancement of cancer and that they might be possible targets for suppressing the development of cancer 5. Taking all of these factors into consideration, it is absolutely necessary to clarify the unique influence that BMs have on the outcomes and therapeutic response of LUAD patients.

Following the initial identification of 37 BM-DEGs by differential analysis and WGCNA, we applied Cox regression analysis to build a prognostic signature based on 4 BMGs (*HMCN2*, *FNLN5*, *ADAMTS15* and *LAD1*). While most of these BMGs are well characterized, the specific mechanisms underlying the downregulation of *HMCN2* expression in cancer cells remain to be investigated 25. Studies have noted that overexpression of fibulin-5 (FBLN5) suppresses DNA synthesis and cyclin A expression in mink lung epithelial cells, thus suppressing tumour cell proliferation 26. Recent studies showed that ladinin-1 (*LAD1*) promoted the proliferation of LUAD cells after cotransfection with circ-ANXA7 knockdown in mammary cells, suggesting that it may be a marker of multiple aggressive tumours^27^, ^28^. In addition, ADAM metallopeptidase with thrombospondin type 1 motif 15 (*ADAMTS15*) has been identified as a crucial component of the Notch signalling pathway 29. The Notch signalling system has been found to be essential for appropriate embryonic development and tissue homeostasis. However, it also plays a crucial role in carcinogenesis and cancer progression 30. Tenascin-C can activate the Notch pathway to promote glioma proliferation by increasing *ADAMTS15* and Jagged1 (*JAG1*) expression 29. The observation that high *ADAMTS15* predicts a poor prognosis in our study could also be attributed to high activity of the Notch pathway triggered by tenascin. The explanation was partly verified by the stronger cell interactions of the tenascin signalling pathway in high-BMG cells from single-cell analysis 31. Taking these factors into account, we can deduce why LUAD patients with high BMG levels have a worse prognosis.

Based on the favourable predictive ability of the BMGs, GO and KEGG enrichment analyses by GSEA were adopted to explore the potential mechanism of BMGs. The enrichment of the gap junction and cadherin binding GO terms in the high-risk group may indicate that cell migration and EMT are triggered, which subsequently promotes tumour metastasis and progression 32, 33. The pro-oncogenic pathways enriched in the high-risk group from KEGG analysis were consistent with the results of GO analysis. In contrast, the enriched functions in low-risk group LUAD comprised activation of mast cells, myeloid cells and platelets, which are mainly associated with immune disease (asthma and systemic lupus erythematosus) 34.

Furthermore, TME analysis (immune infiltration analysis and ESTIMATE) revealed that low-risk LUAD patients had high levels of multiple immune cells (DCs, B cells, CD8+ T cells, etc.) and enrichment of functions essential for antitumour regulation 35. In addition, the differential immune checkpoint expression levels between groups primarily explained the differences in responsiveness to immunotherapy. Notably, the difference is likely mainly attributed to immune cells since the stromal cell scores were comparable between groups.

In the next part, our study focused on ability of BMGs to predict the therapeutic response BMGs. Multiple biomarkers have been developed to identify patients suitable for immunotherapy. It is well established that high TMB is a significant predictor of favourable outcomes in non-small cell lung cancer (NSCLC) 36, 37. TMB was computed as the number of somatic indels and nucleotide substitutions discovered per million bases in the genome's coding region. In addition, the diversity of the TCR repertoire predicting immunotherapy was assessed using the Shannon diversity and richness indexes, which measure the relative abundance and distinctiveness of the TCRs, respectively 16. In our study, high expression of ICI targets in the low-risk group was consistent with the findings regarding TMB and TCR scores in TCGA LUAD samples; all these results indicate that low-risk LUAD patients can benefit from immunotherapy. Additionally, the high risk scores of nonresponsive LUAD patients in both immunotherapy cohorts further corroborated that the low-risk group presents a better response to immunotherapy. This result is also illustrated by the enhanced immune function and increase in inflammatory cell‒cell interactions in low-risk LUADs, as described previously.

Although the BM-related signature was found to be a good indicator of prognosis and ICI response, some limitations still need to be acknowledged. This study involved analysis of public sequencing data and simple in vitro experiments, so additional more complicated in vivo experiments are warranted. In future work, we will continue to explore the role of the signature in specific pathways. Moreover, the immunotherapeutic response based on risk groups was evaluated indirectly, and prospective trials are required to be performed on large-sample patients to explore its predictive reliability.

## Conclusions

In summary, we identified and validated that LUADs with a low BMG signature score had a better prognosis and immunotherapeutic efficiency, which could provide critical guidance for clinical treatment decision making.

## Supplementary Material

Supplementary methods, figures and tables.

## Abbreviations

LUAD: lung adenocarcinoma
TCGA: The Cancer Genome Atlas database
GEO: Gene Expression Omnibus Database
BM-DEGs: basement membrane-related differentially expressed genes
BMGs: BM-related prognostic genes
WGCNA: weighted gene coexpression network analysis
TSNE): TSNE, T-distributed stochastic neighbour embedding
GSEA: gene set enrichment analysis
GSVA: gene set variation analysis
TME: tumor microenvironment analysis
TMB: tumor mutation burden
TCR: T-cell receptor
ICIs: immune checkpoint inhibitors
scRNA: single-cell RNA sequencing analysis
