# Supplementary material for: Comprehensive analysis of the basement membrane in lung adenocarcinoma by bulk and single-cell sequencing analysis
Source: J Cancer. 2023 Jun 4;14(9):1635–47. doi: 10.7150/jca.83407 (PMC10266241; doi:10.7150/jca.83407)
Supplement: Supplementary file 1 — Supplementary methods, figures and tables. [file jcav14p1635s1.pdf]

Content of Supplemental Materials

Detailed Protocols **2**

1. The detailed protocol for scRNA analysis 2

2. The detailed protocols for vitro experiments. 3

The Supplement Tables **5**

Table S1 Baseline characteristics of LUAD patients enrolled in the study. 5

Table S2 List of gene or mRNA names applied in the study. 6

The Supplemental Figures **10**

Figure S1 The scale independence and mean network connectivity of soft-thresholding powers used in WGCNA. 10

Figure S2 Comparisons of BMG-related risk score between age and sex. 10

Figure S3 Survival curves grouped by the risk score and TMB in the TCGA cohort. 11

Figure S4 Heatmap for the inferred copy number each cell. Based on them, the cells were classified into aneuploid(cancer) and diploid cells. 12

Figure S5 Circle plots showed the number of interactions between cells with different BMGs. 12

Figure S6 Circle plots showed the interactions between cancer and other cells with different BMG levels. 13

Figure S7 Circle plots showed the interactions between cancer and other cells with different BMG levels in CD22 and SELL signaling pathway. 13

Reference .... 错误!未定义书签。

Detailed Protocols

1. The detailed protocol for scRNA analysis

The gene-cell matrix was filtered with the following measures (500~20000 detected genes, over 500 UMIs, and less than 10% mitochondrial genes)[21]. The integrated data were first subjected to principal component analysis (PCA) to reduce the dimension by the “RunPCA” function of the “Seurat” package. We selected the first 20 PCs for cell clustering analysis according to the JackStraw analysis and applied the cell clustering analysis by the “FindNeighbors” and “FindClusters” functions with a resolution of 0.5. Then, the T-distributed stochastic neighbor embedding (tSNE) was used to perform clustering using the “RunTSNE ” function. The cell cluster was annotated based on some well-known marker genes from literature and reference data from CellMarker 2.0[38, 39]. Next, we applied CopyKAT with the default parameters to epithelial cells and classified them into aneuploid(cancer) and diploid cells based on the inferred copy number[40].

For cell-cell interaction, gene expression data and assigned cell type were utilized as inputs for analysis [22]. we first applied the "createCellChat" function to create a CellChat object and identified overexpressed ligands or receptors in one cell group. The communication probability was calculated using the "computeCommunProb" function after annotating the object with relevant labels. The "computeCommunProbPathway" function was used to generate cell-cell communications for each cell signaling pathway. Then, the total number and strength of interactions were compared using

“compareInteractions” function. We visualized the different interaction with “netVisual_circle” function and compared the outgoing and incoming interaction strength in 2D space. Finally, we identified overall information flow of the top signaling pathways in each group (“rankNet” function) and compared their association with each cell type (“netVisual_aggregate” function).

2. The detailed protocols for vitro experiments.

The cells were cultured in DMEM containing fetal bovine serum (HyClone, Logan, USA). After cell fusion reached approximately 80%, cells were digested with trypsin at room temperature. The cell liquid was then collected and centrifuged in a centrifuge tube for approximately 5 min, after which the supernatant was discarded. Subsequently, 5 ml of culture medium was added to the cells, followed by gentle blowing and beating to completely suspend the cells, which were then inoculated in a new culture flask. The digested cells were then cultured in a cell incubator with constant temperature and pressure to make the cells adhere to the wall completely.

Total RNA was extracted from selected cells using the TRIzol reagent (CWBIO Co., Ltd.) and reverse transcribed into cDNA using the HiFiScript cDNA Synthesis Kit (CWBIO Co., Ltd.). The SYBR green method was utilized for real-time PCR, which was carried out on an ABI 7500 real-time PCR system. In each group, three identical wells were placed to determine the mean value. The results were analyzed with 7500 System Software V2.3 (Applied Biosystems, California, United States) using beta-actin as the internal standard. The primer sequences of BM-related genes in the model were as follows:

HMCN2

Forwards primer	GAAGCCCAGGTATCGGATAAAG
Reverse primer	TGGGGTTCTCAAATGTTGGGG

FBLN5

Forwards primer AGACGCCCCAAGATTGTTGT

Reverse primer GCACCTGGTTTTGCTTAGCC

ADAMTS15

Forwards primer CGACTGGACCCGGACATTAAC

Reverse primer CCGGCGTCAGGTGTAGGTA

LAD1

Forwards primer CTGCTTCTGAGAGACTACCGA

Reverse primer GATGCTCTGGATGTCCTCGT

For western blots, cells were lysed in RIPA and PMSF (Solarbio Co., Ltd.) and the concentration of protein was measured via BCA method. The protein (20ug/lane) was separated on 10% SDS-PAGE and transferred to PVDF membranes. Then PVDF membranes were blocked with 5% non-fat milk at 37˚C for 2 h and followed by incubation with primary antibody (anti-ADAMTS15 (Abcam Biotechnological Co., Cambridge, UK ab45047); anti-FBLN5(Abcam, ab134136); anti-HMCN2 (Abcam, ab124997); anti-LAD1(Abcam, ab116948) and anti- GAPDH (Abcam ab9485)) 2h at 37˚C or overnight at 4˚C. The blots were then washed with TBST 3 times and incubated with secondary antibodies for 3 h at 37˚C. GAPDH was employed as an endogenous control. Bands were detected using with ECL Plus enhanced chemi-luminescence reagent (Solarbio Co., Ltd.) and quantitated with ImageJ software (version 2.1.4.7 (National Institutes of Health).

The Supplement Tables

Table S1 Baseline characteristics of LUAD patients enrolled in the study. (Table 1 has been updated)

Variable*	Overall<br>N = 1,383	TCGA#<br>N = 521	GSE72094<br>N = 419	GSE68645<br>N = 443
**Age**	67 (60, 74)	66 (59, 73)	70 (64, 76)	65 (58, 72)
Unknown	19	19	0	0
**Sex**				
Female	732 (53%)	280 (54%)	232 (55%)	220 (50%)
Male	651 (47%)	241 (46%)	187 (45%)	223 (50%)
**Stage**				
Stage I	658 (48%)	279 (54%)	265 (64%)	114 (26%)
Stage II	450 (33%)	124 (24%)	69 (17%)	257 (58%)
Stage III	217 (16%)	85 (17%)	63 (15%)	69 (16%)
Stage IV	43 (3.1%)	26 (5.1%)	17 (4.1%)	0 (0%)
Unknown	15	7	5	3
**T**				
T1	322 (34%)	172 (33%)	0 (NA%)	150 (34%)
T2	531 (55%)	280 (54%)	0 (NA%)	251 (57%)
T3	75 (7.8%)	47 (9.1%)	0 (NA%)	28 (6.3%)
T4	31 (3.2%)	19 (3.7%)	0 (NA%)	12 (2.7%)
Unknown	424	3	419	2
**N**				
N0	634 (67%)	335 (66%)	0 (NA%)	299 (68%)
N1	186 (20%)	98 (19%)	0 (NA%)	88 (20%)
N2	128 (13%)	75 (15%)	0 (NA%)	53 (12%)

Variable*	Overall<br>N = 1,383	TCGA#<br>N = 521	GSE72094<br>N = 419	GSE68645<br>N = 443
N3	2 (0.2%)	2 (0.4%)	0 (NA%)	0 (0%)
Unknown	433	11	419	3
**M**				
M0	353 (93%)	353 (93%)	0 (NA%)	0 (NA%)
M1	25 (6.6%)	25 (6.6%)	0 (NA%)	0 (NA%)
Unknown	1,005	143	419	443
**Survival Status**				
Alive	837 (61%)	333 (64%)	297 (71%)	207 (47%)
Dead	546 (39%)	188 (36%)	122 (29%)	236 (53%)
**Survival Days**	852 (500, 1,382)	656 (417, 1,131)	824 (540, 1,012)	1,410 (720, 2,210)
Unknown	31	9	21	1

*The continuous variables are presented as the median (IQR), and the categorical variables are presented as n (%). # Clinical data are available for 521 of 535 patients in the TCGA cohort. Unknown: The number of missing values.

Table S2 List of gene or mRNA names applied in the study.

Type	Gene Names
**BMs-mRNAs**	ACAN, ADAM10, ADAM17, ADAM9, ADAMTS10, ADAMTS13, ADAMTS17, ADAMTS18, ADAMTS2, ADAMTS3, AGRN, AMELX, AMTN, ANG, BGN, CD151, CERT1, COL12A1, COL13A1, COL17A1, COL18A1, COL2A1, COL4A1, COL4A2, COL4A3, COL4A4, COL4A5, COL4A6, COL5A1, COL6A1, COL6A2, COL6A3, COL7A1, COL8A2, COL9A1,

	COL9A2, COL9A3, COLQ, CST3, CTSA, CTSB, CTSD, DAG1, DCC, DCN, DDR2, ECM1, EFEMP1, EFEMP2, FBLN1, FBLN5, FBN1, FBN2, FGF9, FN1, FRAS1, FREM1, FREM2, GPC3, GPC4, GPC6, HMCN1, HSPG2, ITGA2B, ITGA3, ITGA6, ITGA7, ITGA8, ITGB2, ITGB3, ITGB4, ITGB6, LAMA1, LAMA2, LAMA3, LAMA4, LAMB1, LAMB2, LAMB3, LAMC2, LAMC3, LOXL1, MMP1, MMP14, MMP2, MMP21, MPZL2, MUSK, NTN1, P3H1, P3H2, PTPRF, PXDN, ROBO2, ROBO3, ROBO4, RPSA, SERPINF1, SMC3, SMOC1, SMOC2, SPARC, TENM3, TENM4, TGFB1, , TGFB2, TGFBI, TIMP3, TLL1, TNC, USH2A, VCAN, ACHE, ADAMTS1, ADAMTS14, ADAMTS15, ADAMTS16, ADAMTS19, ADAMTS20, ADAMTS4, ADAMTS5, ADAMTS6, ADAMTS7, ADAMTS8, ADAMTS9, BCAN, CCDC80, CD44, COL14A1, COL15A1, COL28A1, COL8A1, CSPG4, DDR1, EGFL6, EGFLAM, EVA1A, EVA1B, EVA1C, FBLN2, FBN3, FMOD, FREM3, GPC1, GPC2, GPC5, HAPLN1, HAPLN2, HMCN2, ISLR, ITGA1, ITGA10, ITGA2, ITGA4, ITGA5, ITGA9, ITGAM, ITGAV, ITGAX, ITGB1, ITGB5, ITGB7, ITGB8, LAD1, LAMA5, LAMB4, LAMC1, LOXL2, LOXL4, LUM, MATN1, MATN2, MATN4, MEGF6, MEGF9, MEP1A, MEP1B, MMP17, MMP26, MMP7, MMRN2, NELL1, NELL2, NID1, NID2, NPNT, NTN4, OGN, OPTC, PAPLN, PHF13, PODN, POSTN, PTN, PXDNL, RECK, ROBO1, SDC1, SDC4, SEMA3B, SLIT1, SLIT2, SLIT3, SPARCL1, SPOCK1, SPOCK2, SPOCK3, SPON1, SPON2, TENM1, TENM2, THBS1, THBS2,

	THBS4, TIMP1, TIMP2, TINAG, TINAGL1, UNC5A, UNC5B, UNC5C, UNC5D, VTN, VWA1
**BMs-DEGs from ifferential analysis**	TINAG, MMP1, ADAMTS20, COL17A1, SMOC1, GPC2, SPOCK1, COL7A1, BCAN, THBS2, OPTC, ADAMTS14, MMP17, ADAMTS19, ACHE, FBN2, COL9A3, ADAMTS16, UNC5D, COL9A1, MMP7, COL5A1, COL9A2, LAD1, ADAMTS18, VTN, VCAN, TENM4, MEP1A, ACAN, LOXL2, TNC, ECM1, POSTN, ITGA2, ITGB4, ITGB8, TIMP1, COL6A3, COL28A1, MMP14, TENM3, LAMB3, GPC6, COL18A1, ITGAV, LOXL1, P3H1, FBN3, COL4A4, COL13A1, GPC5, FBLN1, FRAS1, COL4A3, HMCN2, DCN, ADAMTS15, RECK, TLL1, EFEMP1, NTN4, SEMA3B, ROBO2, LAMC3, MUSK, NPNT, FBLN5, P3H2, SPARCL1, TIMP3, ITGA8, GPC3, OGN, MMRN2, ADAMTS1, SLIT2, SLIT3, DCC, ROBO4, SPOCK2, ADAMTS8, FREM3
**-DEGs from WGCNA**	ADAM10, ADAM17, ADAMTS17, AMELX, CERT1, COL13A1, COL4A3, COL4A4, COL4A5, CTSA, DCC, DCN, DDR2, EFEMP1, FBLN1, FBLN5, FRAS1, FREM2, GPC3, HMCN1, ITGA8, LAMA2, LAMA4, LAMB2, LAMC3, MMP21, MUSK, NTN1, P3H2, ROBO2, ROBO4, RPSA, TGFB2, TIMP3, TLL1, USH2A, ADAMTS1, ADAMTS15, ADAMTS6, ADAMTS8, CD44, DDR1, EGFL6, FBN3, FREM3, HMCN2, ITGA1, ITGA10, ITGA4, ITGA9, ITGAM, ITGAX, ITGB8, LAD1, MEGF6, MEP1A, MEP1B, MMRN2, NELL2, NPNT, NTN4, OGN, RECK, SEMA3B, SLIT2, SLIT3, SPARCL1, SPOCK2, UNC5C, VWA1
**BM-DEGs**	LAD1, MEP1A, ITGB8, FBN3, COL4A4, COL13A1, FBLN1, FRAS1, COL4A3,

	HMCN2, DCN, ADAMTS15, RECK, TLL1, EFEMP1, NTN4, SEMA3B, ROBO2, LAMC3, MUSK, NPNT, FBLN5, P3H2, SPARCL1, TIMP3, ITGA8, GPC3, OGN, MMRN2, ADAMTS1, SLIT2, SLIT3, DCC, ROBO4, SPOCK2, ADAMTS8, FREM3

The Supplemental Figures

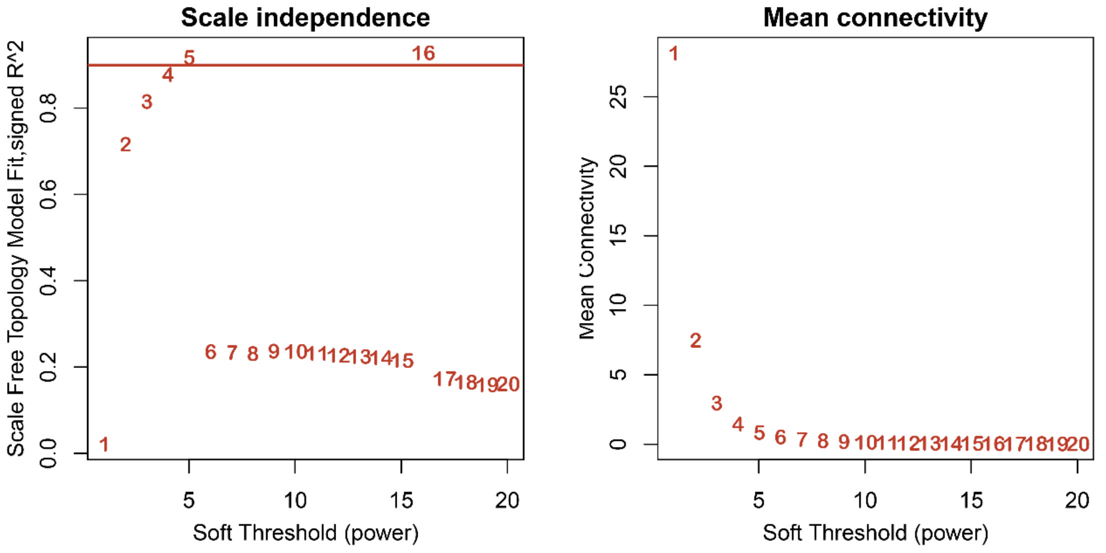

Figure S1 The scale independence and mean network connectivity of soft-thresholding powers used in WGCNA.

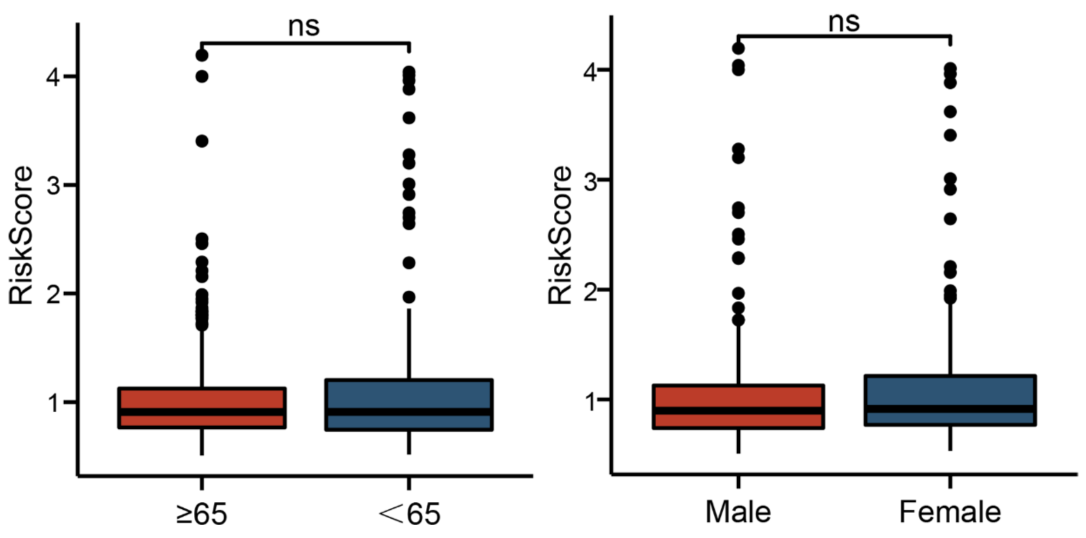

Figure S2 Comparisons of BMG-related risk score between the age and sex groups.

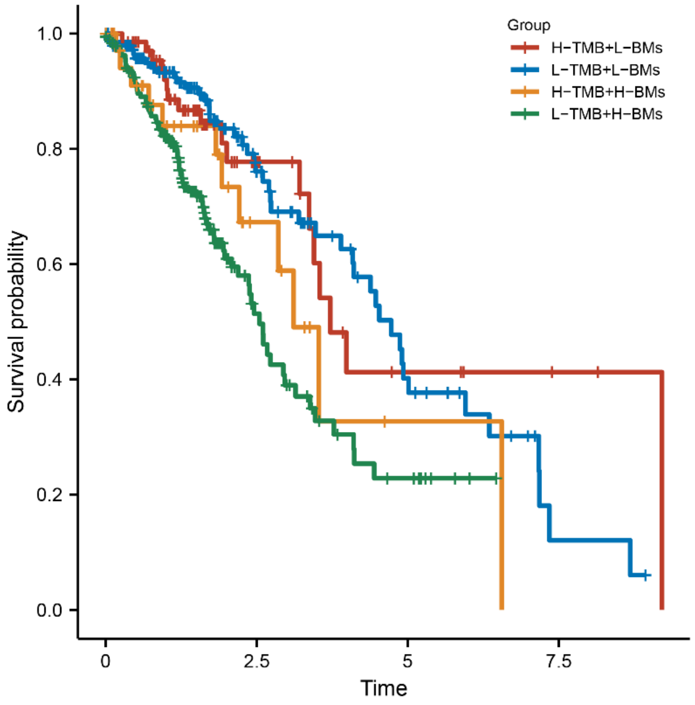

Figure S3 Survival curves grouped by the risk score and TMB in the TCGA cohort.

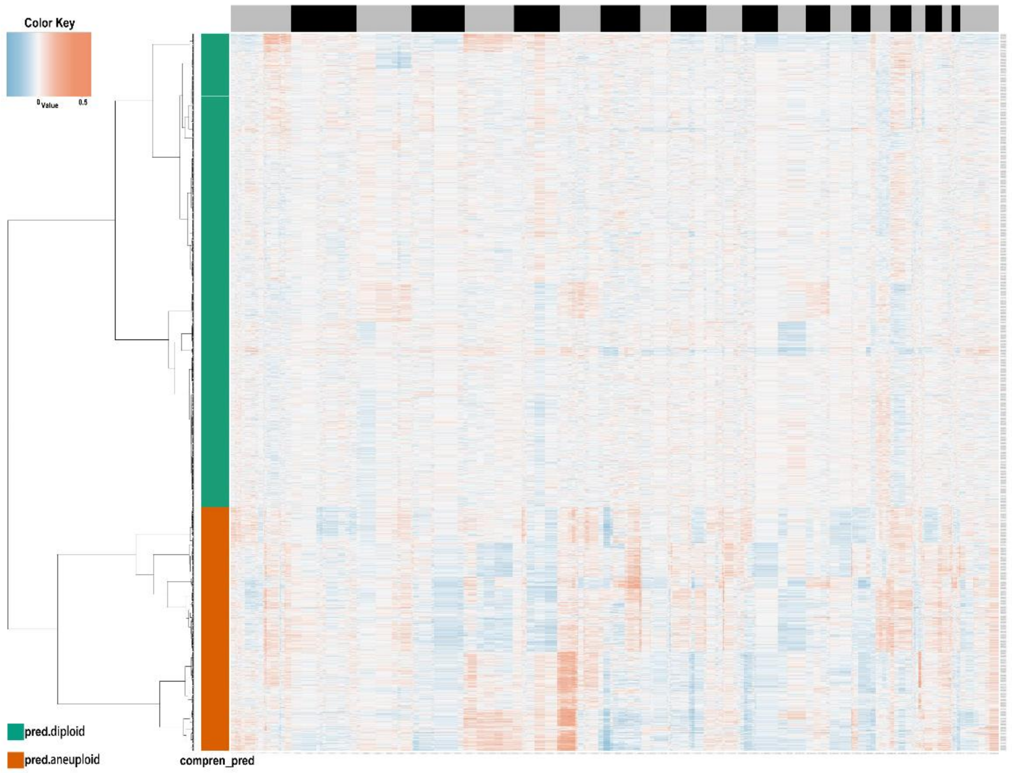

Figure S4 Heatmap for the inferred copy number each cell. Based on them, the cells were classified into aneuploid(cancer) and diploid cells.

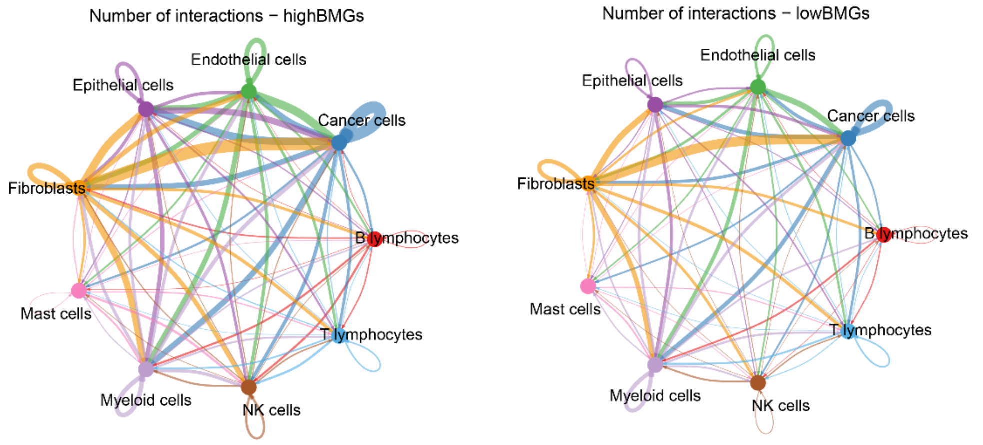

Figure S5 Circle plots showed the number of interactions between cells with different BMGs.

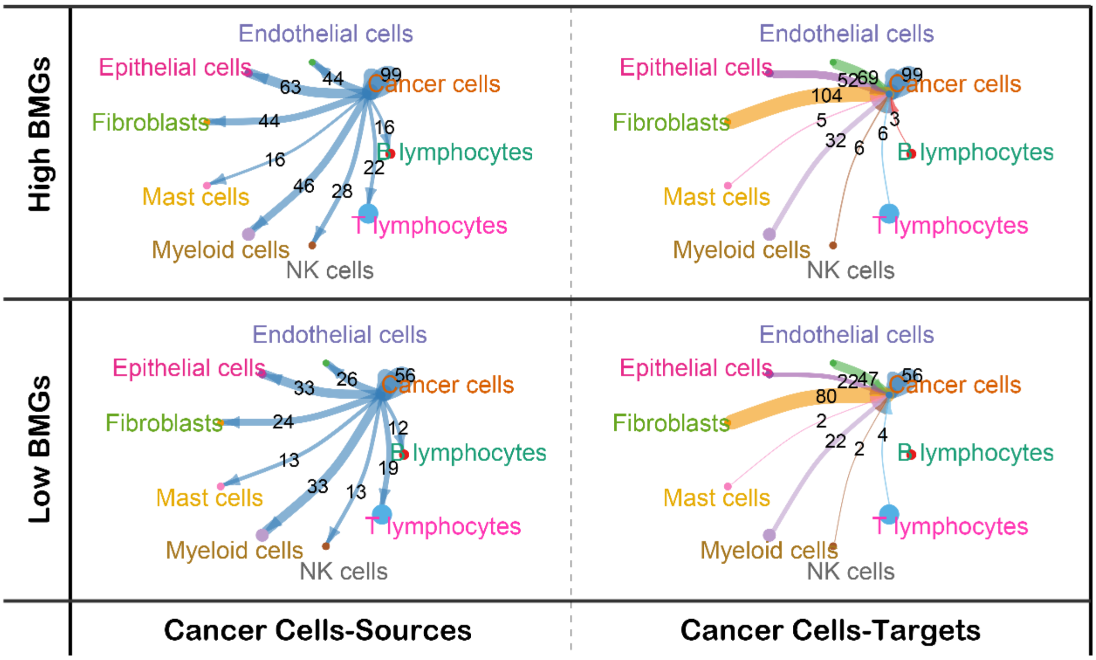

Figure S6 Circle plots showed the interactions between cancer and other cells with different BMG levels

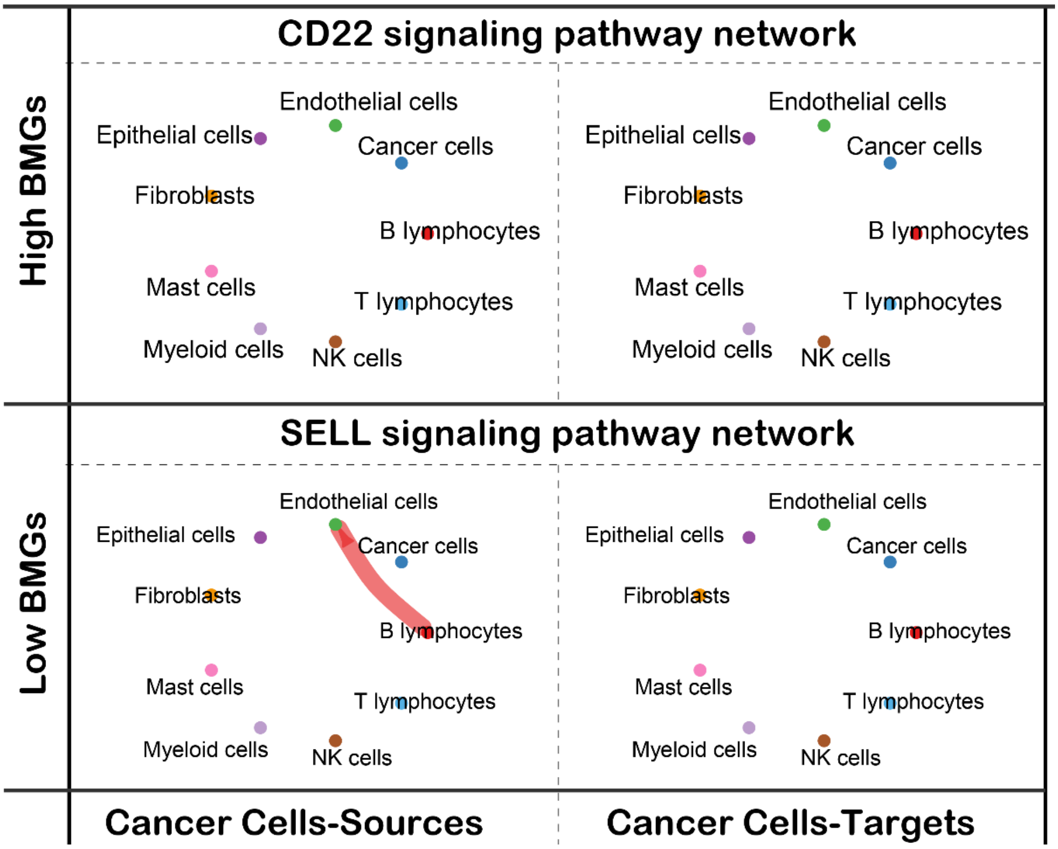

Figure S7 Circle plots showed the interactions between cancer and other cells with different BMG levels in CD22 and SELL signaling pathway.

Reference (Supplement Materials)

1. Hao Y, Hao S, Andersen-Nissen E, Mauck WM, 3rd, Zheng S, Butler A, Lee MJ, Wilk AJ, Darby C, Zager M *et al*: Integrated analysis of multimodal single-cell data. *Cell* 2021, 184(13):3573-3587.e3529.
2. Hu C, Li T, Xu Y, Zhang X, Li F, Bai J, Chen J, Jiang W, Yang K, Ou Q *et al*: CellMarker 2.0: an updated database of manually curated cell markers in human/mouse and web tools based on scRNA-seq data. *Nucleic acids research* 2022.
3. Kim N, Kim HK, Lee K, Hong Y, Cho JH, Choi JW, Lee JI, Suh YL, Ku BM,

Eum HH *et al*: Single-cell RNA sequencing demonstrates the molecular and cellular reprogramming of metastatic lung adenocarcinoma. *Nature communications* 2020, 11(1):2285.

4. Gao R, Bai S, Henderson YC, Lin Y, Schalck A, Yan Y, Kumar T, Hu M, Sei E, Davis A *et al*: Delineating copy number and clonal substructure in human tumors from single-cell transcriptomes. *Nature biotechnology* 2021, 39(5):599-608.
5. Jin S, Guerrero-Juarez CF, Zhang L, Chang I, Ramos R, Kuan CH, Myung P, Plikus MV, Nie Q: Inference and analysis of cell-cell communication using CellChat. *Nature communications* 2021, 12(1):1088.
